# Fertility Sparing Treatment in Gastric-Type Endocervical Carcinoma

**DOI:** 10.3390/cancers13205177

**Published:** 2021-10-15

**Authors:** Agnieszka Rychlik, Denis Querleu, Mariusz Bidzinski

**Affiliations:** 1 Department of Gynaecologic Oncology, National Research Institute of Oncology, 02-781 Warsaw, Poland; Mariusz.Bidzinski@pib-nio.pl; 2 Department of Obstetrics and Gynecology, University of Strasbourg, 67081 Strasbourg, France; denis.querleu@esgo.org; 3 Division of Gynecologic Oncology, Policlinico Agostino Gemelli, 00168 Rome, Italy

**Keywords:** fertility sparing management, cervical cancer, gastric-type cervical cancer

## Abstract

**Simple Summary:**

Due to a trend toward late childbearing, fertility preservation has become a major issue in young patients with gynecological cancer. Surgical fertility sparing management is universally acknowledged as an acceptable option in eligible patients with cervical cancer. Excisional cone biopsy or trachelectomy are now standard surgical procedures offered to selected patients with gynecological malignancies who wish to preserve their fertility. Neoadjuvant chemotherapy followed by surgery is another therapeutic option studied currently in numerous clinical trials. However, fertility preserving treatment is generally not recommended in rare histological types of cervical cancer, including clinically aggressive gastric-type endocervical carcinoma. Due to controversies in this emerging topic, a review of literature and international database was carried out, in search of solid evidence on fertility sparing management in gastric-type endocervical carcinoma.

**Abstract:**

Fertility sparing management of cancer is one of the main components of quality-of-life issues. Early-stage cervical cancer, frequently diagnosed in women of reproductive age, can potentially be treated conservatively. However, some rare histological types of cervical cancer present with aggressive clinical behavior. Particularly, in the newly introduced concept of gastric-type endocervical carcinoma, fertility sparing management is ‘a priori’ not recommended. Even so, this issue remains undocumented. For this reason, a selected review of the current literature on gastric type endocervical carcinoma was carried out through PubMed. The keywords included “gastric-type cervical cancer”, “gastric-type endocervical carcinoma”, “conservative surgery”, “conservative treatment”, “fertility sparing surgery”, “radical trachelectomy”, “laparoscopic trachelectomy”, “robotic trachelectomy”, “laparotomic trachelectomy”, “abdominal trachelectomy”, “trachelectomy”, “neoadjuvant chemotherapy”, “conisation”, and “cone resection”. A search in the European Network on Cancer, Infertility and Pregnancy (INCIP) database was performed. The rarity of gastric-type endocervical carcinoma does not allow for conclusions on fertility sparing management with solid evidence. However, diffuse character of the disease and aggressive clinical behavior contraindicate a conservative treatment in young women with gastric type cervical cancer.

## 1. Introduction

Cancer survival has improved over the last years. From 2002 to 2012, there was an 83% 5-year survival rate within the group of women younger than 45 years diagnosed with cancer [1]. In consequence, there are many young survivors of reproductive age. According to the National Cancer Institute data, there are around 250,000 cancer survivors aged 20 to 39 years [2]. One in 51 women will be diagnosed with cancer by age 39, and 1 in 19 women by age 49 [3,4].

Fertility preservation and other quality of life issues have become important matters to consider in the management of these patients. Oncofertility has become an important component of cancer care. It places a special importance on the psychological aspect of infertility related to cancer treatment and helps patients to make conscious decisions regarding cancer treatment and fertility, with the objective to reconcile the objective of curing cancer with a hope of maternity.

All specialists involved in the care of oncological patients should be informed about the possibilities of fertility preservation. This is especially important true in patients with gynecological cancer. According to the American Society of Clinical Oncology (ASCO) guidelines: “Doctors should be prepared to discuss the effects of cancer treatment on fertility infertility as a potential risk of therapy and provide appropriate referrals to reproductive specialists when indicated. This discussion should take place as soon as possible once a cancer diagnosis is made and before a treatment plan is formulated [5].” Unfortunately, female cancer patients do not universally receive counselling on the effects of treatment on fertility and fertility preservation.

Cervical cancer is one of the top three cancers affecting women younger than 45 [6]. Fertility sparing management is, then, an emerging issue in cervical cancer treatment. According to European guidelines, conservative treatment “should be considered for every woman with a desire to spare fertility and is diagnosed of histologically proven squamous cell carcinoma or usual-type, Human Papilloma Virus (HPV)-related adenocarcinoma equal to or less than 2 cm of the largest diameter” [7]. For properly selected patients, less radical surgery can be considered. In larger tumors (>2 cm), neoadjuvant chemotherapy followed by surgery can be an alternative. The benefit of tumor downsizing with regard to prognosis is considered as controversial. However, it has been documented in numerous retrospective studies [8,9] and is being investigated in prospective trials, NCT00039338, NCT04016389 [10,11].

However, fertility-sparing treatment is not recommended in current guidelines for uncommon histological types of cervical cancer including non-HPV-related gastric-type adenocarcinoma, which tend to exhibit aggressive behavior. [7,12]. This review was carried out to document this general recommendation.

## 2. Materials and Methods

In order to conclude about the recommendation, a selected review of the current literature on gastric type endocervical carcinoma was performed through MEDLINE, Current Contents, and PubMed. Terms included “gastric-type cervical cancer”, “gastric-type endocervical carcinoma”, “conservative surgery”, “conservative treatment”, “fertility sparing surgery”, “trachelectomy”,“radical trachelectomy”, “laparoscopic trachelectomy”, “laparotomic trachelectomy”, “robotic trachelectomy”,“abdominal trachelectomy”, “neoadjuvant chemotherapy”, “conisation”, and “cone resection”. Subsequently, a search in the European Network on Cancer, Infertility and Pregnancy (INCIP) database was performed. INCIP is an European Society of Gynaecologic Oncology network. The primary objective of INCIP is to establish an international registry on cancer during pregnancy and fertility preservation during cancer treatment. Data refer to the oncological, obstetrical, and neonatal settings. For fertility preserving methods, all cancer types and treatment modalities are included.

## 3. Result

In spite of a thorough search, not a single paper focused on reporting on attempts at fertility preserving management in gastric type cervical cancer. Moreover, no record of gastric-type carcinoma and fertility sparing treatment was found in the large INCIP database. We are, then, in an unusual situation in which the literature cannot answer adequately the question of the feasibility, safety, and outcomes of fertility preserving surgery, with or without neoadjuvant chemotherapy in this particular histological subtype. Consequently, the current guidelines are not based on any level of evidence. This justifies a comprehensive discussion in order to find arguments in order to document the informed consent at the time of offering the young gastric-type cancer patients desiring fertility a radical management.

## 4. Discussion

### 4.1. Specificity of Gastric-Type Endocervical Adenocarcinoma in the Context of Fertility Sparing Surgery

#### 4.1.1. Epidemiology

The exact incidence of gastric type endocervical carcinoma is difficult to assess. A large multinational European study suggests that the gastric type represents 1.5% of cervical adenocarcinoma cases [13], whereas in Japan the incidence is much higher, estimated to be 25–30% of all endocervical adenocarcinomas [14,15]. The lack of data in Europe is then explained by the rarity of the disease. It is hoped that investigations in Japan will clarify the issues that are pertinent to fertility preservation.

#### 4.1.2. Pathologic Features of Gastric Type Endocervical Carcinoma

Gastric-type cervical adenocarcinoma together with the new classification of invasive adenocarcinomas of the endocervix was introduced in the World Health Organization (WHO) classification in 2014 in the 4th edition of the WHO classification for tumors of the female genital tract [16].

In 2017, an international panel proposed the International Endocervical Adenocarcinoma Criteria and Classification (IECC) system that separates endocervical carcinomas into two major groups—those that are human papillomavirus-associated and those that are non-HPV-associated—based on morphology (linked to etiology) alone. The major subtype of the latter category is gastric-type endocervical carcinoma [17].

Gastric-type endocervical carcinoma is not a single entity, but rather a spectrum of lesions. The spectrum includes well-differentiated minimal deviation adenocarcinoma of mucinous type (formerly known as adenoma malignum). Adenoma malignum is characterized by benign-appearing, irregularly shaped glands that often deeply infiltrate cervical stroma. It also includes moderately to poorly differentiated ECA containing gastric-type mucin.

The current concept of gastric-type endocervical carcinoma was introduced in 2007 by Kojima et al. It was first defined as a subtype of mucinous carcinoma characterized by cells with voluminous clear eosinophilic cytoplasm and distinct cell borders [14]. It has been demonstrated that this morphologically defined variant of endocervical adenocarcinoma invariably shows a gastric immunophenotype: positive for HIK1083 (mouse monoclonal; KantoKagaku, Tokyo, Japan) and/or MUC6 expression (mouse monoclonal, CLH5; Novocastra Laboratories, Newcastle, UK). The diagnostic criteria were validated in other series and the interobserver reproducibility is high. Gastric-type adenocarcinoma seems to be a completely different type of endocervical carcinoma, which is characterized by distinctive morphological criteria [18].

It has been proposed that gastric-type endocervical carcinoma arises from precursor benign lesion-lobular endocervical glandular hyperplasia/pyloric gland metaplasia (LEGH/PGM), which is characterized by multiple cysts commonly located in the upper portion of the cervix that histologically expresses gastric immunophenotype [19,20].

Clinically gastric-type endocervical carcinoma is defined by characteristic features. When compared with usual type HPV-related endocervical carcinoma, gastric-type endocervical carcinomas are usually more extensive in size. The FIGO stage at diagnosis is higher. The proximal endocervix seems to be a common site of origin and, therefore, detection at early stages is challenging [21]. The PAP smear tests do not show abnormalities in the majority of cases [22].

Consequently, several features of the gastric-type endocervical adenocarcinoma are not favorable for fertility sparing surgery, even using the most radical abdominal trachelectomy: (1) involvement of the entire endocervix, (2) frequent deep stromal invasion, (3) extensive precancerous lesions, located in the upper portion of the cervix, with difficult differential diagnosis. For these reasons, achieving clear exocervical, endocervical, and stromal margins is extremely difficult without removing the entire endocervix, which results in extremely poor fertility and obstetrical outcomes.

#### 4.1.3. Radiologic Features

As described by Kido et al., gastric-type endocervical carcinomas show distinctive radiological features. At Magnetic Resonance Imaging (MRI), gastric-type endocervical carcinoma presents as an infiltrating multicystic mass with solid portions of endophytic growth, located usually in the upper cervical canal, and with tiny cysts [23]. In this series, 14 of the 15 cases of gastric-type endocervical carcinoma were located in the higher portion of the cervical canal or involved the entire cervix. The majority of cases also showed parametrial, endometrial, and vaginal involvement; however, the radiological diagnosis of these features is often underestimated.

In the publication by Park et al. gastric-type mucinous adenocarcinoma lesions showed an infiltrative shape without a barrel-shaped appearance in the majority of patients [24].

The characteristic multicystic pattern is described as a multicystic mass with solid portions composed of microscopic cysts. It can be present not only in gastric-type endocervical carcinoma, but is also typical for lobular endocervical glandular hyperplasia (located usually in the internal os of the cervix) and other cystic lesions, which makes the differential diagnosis difficult.

**Figure 1:**
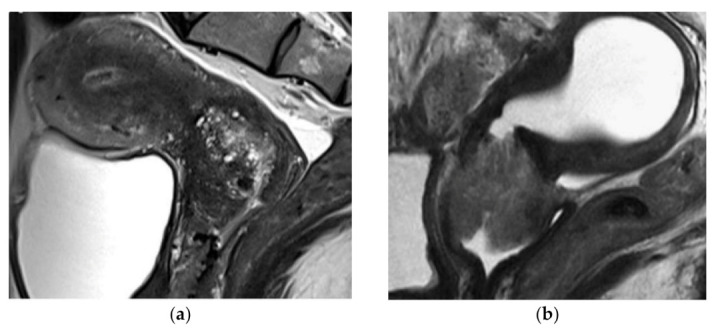
(**a**,**b**) Magnetic Resonance Imaging of gastric-type endocervical carcinoma. Reprinted with permission from Ref. [25] Copyright 2018 by the BMJ Publishing Group Ltd.

Examples of characteristic magnetic resonance images are presented in Figure 1 [25].

Therefore, the radiologic features confirm the microscopic data, and included characteristics generally considered as contra-indications to fertility surgery in the common HPV-related type cervical cancer: large size, stromal invasion, involvement of the entire endocervix up to the internal os.

#### 4.1.4. Prognosis

The emerging definition of gastric-type endocervical carcinoma of the cervix has been accepted worldwide because of the negativity for human papillomavirus and its aggressive clinical behavior. However, there are only a few publications that consider the prognosis of gastric-type endocervical carcinoma.

In the original article by Kojima et al. [14], 5-year disease-free survival and 5-year disease-specific survival of patients with gastric-type adenocarcinoma were significantly poorer when compared with other histological types, 25% vs. 75%; *p* = 0.0001 and 30% vs. 77%; *p* < 0.0001, respectively.

Later, Karamuzin et al. revised the database from the Memorial Sloan Kettering Cancer Center together with two collaborating institutions. The authors found significantly worse survival outcomes for gastric-type adenocarcinoma when compared with usual-type adenocarcinoma, similar to the data presented by Kojima’s group. In this series, the majority of patients presented at an advanced stage and pelvic, abdominal, and distant metastases were not uncommon [26].

Furthermore, Kojima et al. demonstrated that there is a significant difference in chemosensitivity and survival outcome between the usual-type adenocarcinoma and gastric-type adenocarcinoma. The response rate of gastric-type adenocarcinoma to neoadjuvant chemotherapy consisting of docetaxel and carboplatin was significantly lower, resulting in worse survival outcomes [15].

Consequently, clinicians are entitled to be reluctant to propose a fertility sparing management, based on tailored surgery, in a locally aggressive tumor. In addition, the poor chemosensitivity is not favorable to the use of neoadjuvant chemotherapy to overcome the generally large size of the tumor.

### 4.2. Techniques and Indications of Fertility Sparing Surgery in Cervical Cancer

#### 4.2.1. Principles and Results of Dargent Radical Trachelectomy

The concept of fertility sparing surgery in early-stage cervical cancer has been proposed by Daniel Dargent in 1994 [27,28,29], historically preceded by Aburel (abdominal radical trachelectomy) [30].

The original technique of vaginal radical trachelectomy was described as a modification of the Schauta-Stoeckel operation. The procedure begins with a creation of a “vaginal cuff,” in order to ensure an adequate vaginal margin of approximately 1 to 2 cm, to prevent contamination of the operating field by cancer cells and to firmly pull down the cervical area. Several Kocher Forceps are placed on the vagina around the cervix. Adrenaline solution is injected under the vaginal mucosa to reduce bleeding and facilitate the vaginal dissection. The ventral and dorsal parts of incised vaginal mucosa are approximated using Krobach or similar clamps placed horizontally. The Douglas pouch is then opened. The rectovaginal septum is developed. The pararectal fossas are opened. The rectouterine ligaments are transected. In the later description of the technique, the opening of the Douglas pouch was not necessary. Cervix is then pulled down along with the vaginal cuff using traction on the Krobach forceps. The vesicovaginal and vesicouterine septums are opened and widely developed by blunt dissection. At this moment, the most crucial part of the procedure, the location of the ureters, can be undertaken. The paravesical spaces are widely opened. The ureters are now localized within the bladder pillars, a surgical structure delineated by the vesicouterine space medially and the paravesical space laterally. When the prevesical and paravesical spaces are developed, the uterine artery can be seen and the ureter knee can be palpated then dissected within the mid-portion of the bladder pillar. At this time, the ventral and dorsal part of paracervical ligaments can be transected between two clamps or using a bipolar energy instrument. The lateral margin is located 2 cm from the vaginal cuff. This corresponds to the current class-B radicality according to the Querleu-Morrow classification. However, a less radical (type A) surgery can be performed in low-volume disease with minimal risk of paracervical involvement.

**Figure 2:**
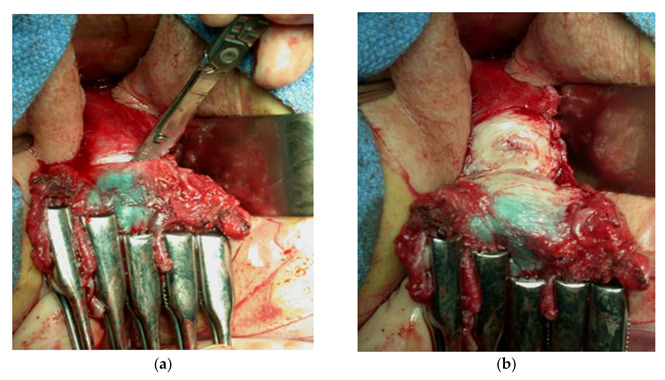
(**a**,**b**) Intraoperative images of radical trachelectomy (Courtesy Michel Roy).

Whatever the radicality, only the descending branch of the uterine artery, the cervicovaginal branch, is coagulated or ligated. The uterine artery is routinely preserved -even though unwanted division does not result in significant alteration of the main blood supply to the uterus. The final step of the surgical intervention consists of transection of the cervix that is divided 5 mm under the isthmus. The ideal margin 1 cm above the upper limit of the tumor, but there is no evidence of the literature supporting the need of a wide margin. Currently, obtention of a clear margin is the only requirement. Cervical cerclage is placed around the isthmus [30,31]. The Douglas pouch is closed. The cervical stump is approximated using interrupted sutures to the vaginal mucosa, then a cervicovaginal anastomosis is completed, taking care not to cover the cervical canal. Intraoperative images of radical vaginal hysterectomy are presented in a Figure 2.

From the first publication of vaginal radical trachelectomy, there have been more than 1000 cases reported in the literature. Accumulating data confirm that radical trachelectomy is oncologically safe and the obstetrical results are encouraging. Since 1994, more than 300 live births have been reported in the literature [32]. Laparoscopic, robot-assisted laparoscopic, and abdominal variants have also been described.

It is greatly important to adequately select patients for the procedure. The adherence to the selection criteria should be strict. Current criteria accepted by the international societies for radical trachelectomy are as follows: (1) patient that strongly desires to preserve her fertility; (2) typical histopathology (squamous cell carcinoma, adenocarcinoma, or adenosquamous) should be confirmed, rare histopathology subtypes are not eligible; (3) International Federation of Gynaecology and Obstetrics (FIGO) stage IA1 with lymphovascular space invasion (LVSI), FIGO IA2 or IB1; (4) no lymph node metastasis; tumor localization that allows achieving clear margins. These characteristics correspond to the current description of early-stage cervical cancers that can be surgically managed with less radical surgery [7,12]. The LVSI is not an absolute contraindication of fertility sparing surgery; however, it is considered as a risk factor of recurrence.

Magnetic resonance imaging or an expert ultrasound are obligatory to correctly assess the location and size of the disease. The preoperative workup frequently includes a diagnostic cone biopsy.

The original Dargent’s technique applied two concepts: the combination of laparoscopic lymph node evaluation and transvaginal approaches and the margin concept.

Pelvic lymph node assessment is an obligatory step to exclude pelvic metastases. Any suspicious peritoneal lesion, enlarged lymph node or ovarian mass must be resected and assessed on frozen section. In case of proven metastatic disease, the procedure should be abandoned. Sentinel lymph node biopsy improves the sensitivity of the procedure, while permitting identification of atypical lymphatic drainage [33]. The ultrastaging of sentinel lymph node detects low-volume metastasis. Frozen section examination of the sentinel lymph node is an acceptable technique; however, it should only be performed by an expert pathologist [34]. The risk of a false-negative frozen section is not negligible, especially in low-volume disease [35]. If positive lymph nodes are identified, the fertility sparing intervention must be abandoned. Para-aortic lymphadenectomy up to the level of inferior mesenteric artery is recommended in case of pelvic lymph node positivity.

In the original technique, a frozen section examination is also systematically performed on the endocervical margin. The ideal upper endocervical margin has been described as 8–10 mm, with a minimal 5 mm margin required. The remining endocervix should not be less than 5 mm considering the risk of premature delivery [36].

In case of positive upper margin or residual endocervix length shorter than 5 mm, a radical hysterectomy is indicated. The patient should always be informed about this eventuality before surgery.

Available literature suggests that the recurrence rate after radical trachelectomy is less than 5% and mortality rate less than 2% [37]. In an earlier review, tumor recurrence rate was 4.2–5.3% with a mortality rate 2.5–3.2%, and was not superior to adverse outcomes observed after radical hysterectomy [38].

A review by Bentivegna et al. identified and included 1364 patients submitted to radical vaginal trachelectomy. Twenty-four patients (1.5%) died of disease, and 58 patients (3.8%) experienced recurrence [39]. According to the available data, uterine-preserving surgery does not seem to compromise oncologic outcomes in a select group of young women with early-stage cervical cancer [40].

#### 4.2.2. Other Approaches of Radical Trachelectomy

Radical trachelectomy can be performed abdominally by an open technique, or laparoscopically, with or without robotic assistance [41,42,43,44,45]. Compared with the vaginal approach, the abdominal radical trachelectomy was initially thought to be more radical as for the parametrial and paracervical dissection. It is more consistent with a Querleu-Morrow type-C resection. A larger portion of the parametria is included in the resection and uterine arteries are often interrupted at their origin from the internal iliac artery. That is why it was offered to patients with unfavorable histopathological factors, larger tumors, with LVSI and/or deep stromal infiltration. The technique of abdominal radical trachelectomy mimics the steps of radical abdominal hysterectomy, and therefore does not require any specific training for an experienced gynecologic oncologist. The main issue in regard to the abdominal technique is related to its impact on obstetrical outcomes. The preservation of the uterine arteries is technically possible; however, its benefit is not clear. The fertility outcomes of the specific techniques are discussed below.

The laparoscopic or robotic-assisted operation is similar to the laparotomic trachelectomy. The oncological safety of the minimally invasive approaches is currently challenged with the results of the LACC trial [46]. However, the first results from the largest retrospective international collaboration study, International Radical Trachelectomy Assessment (IRTA-study), have shown similar progression-free and overall survival for patients with tumors up to 2 cm undergoing open vs. minimally invasive radical trachelectomy [47].

#### 4.2.3. Obstetrical Outcomes

As far as obstetrical outcomes are concerned, a recent review identified 2777 patients submitted to fertility sparing surgery. There were 944 pregnancies. The overall fertility rate was 55%. The pregnancy rate was higher in patients submitted to a vaginal 57% (241 of 424) or minimally invasive 65% (57 of 87) radical trachelectomy compared with a laparotomic radical trachelectomy 44% (135 of 310) vs. and (*p* < 0.001). The live birth rate was similar in all the fertility sparing techniques, around 70%. There was no difference in pregnancy rates regardless of the ligation or preservation of the uterine artery. Risk of preterm delivery ranged from 39% for the Dargent’s procedure and 57% for laparotomic radical trachelectomy. There was no difference in life births rate between the techniques [48]. In other systematic reviews, second trimester miscarriage was twice more frequent than in a general population, but only 12% with significant prematurity [49]. Infertility is observed in 30% of patients due to cervical factors, such as cervical stenosis, decreased cervical mucus, surgical adhesion formation, and subclinical salpingitis [50]. Morbidity associated with radical vaginal trachelectomy is low [32].

#### 4.2.4. Simple Trachelectomy or Cone Resection

Excisional cone biopsy or simple trachelectomy is an acceptable fertility sparing surgical technique for patients with FIGO stage IA1-IB1 cervical cancer without deep stromal invasion.

The need of parametrectomy has been discussed in various retrospective trials [51,52] and is being addressed in a prospective phase 3 randomized SHAPE trial (NCT02213861) [53].

The systematic review by Bentivegna et al. found 13 series or case reports addressing simple trachelectomy or conization in 242 patients with stage IB1 tumors of 2 cm or smaller. The authors concluded that cone resection or simple trachelectomy can be indicated for Stage IB1 < 2 cm LVSI negative patients, however, the safety of omitting the parametrectomy in patients with positive LVSI has to be assessed in further studies [39].

#### 4.2.5. Neoadjuvant Chemotherapy Followed by Fertility Sparing Surgery

In larger tumors associated with negative prognostic factors such as deep stromal invasion, upfront trachelectomy is not recommended.

In these cases, in patients who wish to preserve fertility, neoadjuvant chemotherapy can be proposed. The feasibility of this therapeutic strategy was first described by Kobayashi et al. and later by Plante et al. in three patients with lesions measuring from 3–4 cm who showed an excellent response to neoadjuvant chemotherapy that was followed by radical trachelectomy [54,55].

Neoadjuvant chemotherapy can potentially improve surgical margins and enable less radical endocervical tissue resection and better obstetrical outcomes [56,57]. This strategy is only acceptable assuming a comprehensive diagnostic workup and surgical staging discarding nodal or metastatic disease. Extensive counseling regarding the side effects and risks of chemotherapy is essential.

The most frequently used neoadjuvant regimen is three courses of platinum-based chemotherapy. As far as the surgical technique is concerned, either radical trachelectomy or simple trachelectomy/conization are used. In a recent systematic review among patients who received initial chemotherapy, 42 underwent radical trachelectomy and 51 underwent simple trachelectomy, cold-knife, or laser conization [26].

An interesting publication from the group from Leuven reported 10 patients with FIGO stage IB1 and one patient with IB2 cervical cancer managed with neoadjuvant chemotherapy and conization. Paclitaxel–ifosfamide–carboplatin or a combination of paclitaxel–carboplatin was used in this study. Complete response after chemotherapy was reported in 64% of patients. Patients with residual disease were treated by radical hysterectomy [58].

The repose to neoadjuvant chemotherapy is a strong independent predictor of survival. Poor chemotherapy response is an absolute indicator of the need of a definitive multimodal management without fertility preservation.

Safety of this therapeutic strategy has been addressed in various retrospective series [59,60]. In a recent meta-analysis by van de Kol et al., studies on abdominal radical trachelectomy and neo-adjuvant chemotherapy followed by vaginal radical trachelectomy were evaluated. The chances for recurrence were not statistically different between the two groups. However, the pregnancy rate was significantly different: 70% in the neo-adjuvant chemotherapy followed by vaginal radical trachelectomy group and only 21% in the abdominal radical trachelectomy group [24]. A Recently opened CONTESSA/NEOCON prospective observational trial was designed to give robust data on efficacy and safety of neoadjuvant chemotherapy followed by less radical surgery [11]. The clinical incentive of the study was to improve obstetrical outcomes as compared with abdominal radical trachelectomy without hampering of oncological outcomes as compared with radical hysterectomy.

### 4.3. Available Recommendations and Guidelines in Gastric Type Cervical Cancer

The European guidelines do not recommend fertility sparing management in patients diagnosed of rare histological subtypes, including non-HPV-related adenocarcinomas (except for adenoid basal carcinoma), which tend to exhibit aggressive behavior [7]. The National Comprehensive Cancer Network (NCCN) guidelines also advise against fertility sparing treatment for small-cell neuroendocrine tumors and gastric-type endocervical carcinoma [13]. These recommendations are motivated, on one hand, by a proximal, endocervical origin of gastric-type carcinoma of large size with a technical difficulty to achieve an optimal endocervical margin, and, on the other hand, the aggressive clinical behavior of gastric-type endocervical carcinoma that impacts survival. In addition, the high endocervical location of precancerous lesions would require a complete resection of the endocervix, which is associated with low fertility and high rate of pregnancy loss. However, the clinician must be aware of reports on fertility preservation in another aggressive cervical cancer histologic subtype, neuroendocrine carcinoma [61].

The potential risk of ovarian metastases does not even allow the consideration of ovarian preservation. As a consequence, in a fertility preserving mindset, and depending, oocyte retrieval and embryo cryopreservation (if the patient has a stable conjugal relationship) or oocyte vitrification (if the patient is single) should be offered in order to keep open the possibility of obtaining pregnancy via surrogacy. In studies on breast cancer patients, the time from referral to a reproductive endocrinologist to oocyte retrieval did not result in a significant delay in the start of cancer treatment [62]. Consultation prior to a radical treatment, either surgery or pelvic irradiation, should be considered and the patient should be aware of the possibility of gestational carrier pregnancy. Physicians who are counseling about fertility preservation options need to include surrogacy in the conversation. In countries where surrogacy is not legal, patients may have to resort to surrogacy tourism [63].

Uterine transplant, an innovative technique in the field of reproductive medicine, has showed a remarkable successful outcome in patients with congenital Müllerian malformations, such as in the Mayer–Rokitansky–Küster–Hauser syndrome, or more commonly acquired uterine infertility (Asherman’s syndrome, myomas, hysterectomies) [64]. Hopefully, in the future, this technique could be adapted for gynecological cancer survivors.

## 5. Conclusions

Even in the absence of literature documenting the oncological and obstetrical outcomes of fertility sparing surgery, there are many arguments against the fertility sparing management. The lack of relevant literature is related to the rarity of the disease and to the reluctance of the clinician to offer fertility sparing management. Due to diagnostic challenges, aggressive nature and chemoresistance conservative surgery or neoadjuvant chemotherapy remain not recommended for treating gastric-type endocervical adenocarcinoma, including minimal deviation adenocarcinoma. The experimental attempts of fertility sparing management in gastric-type endocervical carcinoma should be limited to highly selected patients, prospectively monitored, and performed under an informed consent.

## Data Availability

The data presented in this study are available on request from the corresponding author.
